# Modeling and Simulation of 3D Food Printing Systems—Scope, Advances, and Challenges

**DOI:** 10.3390/foods12183412

**Published:** 2023-09-13

**Authors:** Vijayakumar Raja, Shubham Nimbkar, Jeyan Arthur Moses, Sinija Vadakkepulppara Ramachandran Nair, Chinnaswamy Anandharamakrishnan

**Affiliations:** 1 Food Processing Business Incubation Centre, National Institute of Food Technology, Entrepreneurship and Management—Thanjavur, Ministry of Food Processing Industries, Government of India, Thanjavur 613005, Tamil Nadu, India; 2 Computational Modeling and Nanoscale Processing Unit, National Institute of Food Technology, Entrepreneurship and Management—Thanjavur, Ministry of Food Processing Industries, Government of India, Thanjavur 613005, Tamil Nadu, India; 3 CSIR—National Institute for Interdisciplinary Science and Technology (NIIST), Ministry of Science and Technology—Government of India, Thiruvananthapuram 695019, Kerala, India

**Keywords:** modeling, computational modeling, 3D food printing, AI and 3D printing, simulation, 3D printing optimization

## Abstract

Food 3D printing is a computer-aided additive manufacturing technology that can transform foods into intricate customized forms. In the past decade, this field has phenomenally advanced and one pressing need is the development of strategies to support process optimization. Among different approaches, a range of modeling methods have been explored to simulate 3D printing processes. This review details the concepts of various modeling techniques considered for simulating 3D printing processes and their application range. Most modeling studies majorly focus on predicting the mechanical behavior of the material supply, modifying the internal texture of printed constructs, and assessing the post-printing stability. The approach can also be used to simulate the dynamics of 3D printing processes, in turn, assisting the design of 3D printers based on material composition, properties, and printing conditions. While most existing works are associated with extrusion-based 3D printing, this article presents scope for expanding avenues with prominent research and commercial interest. The article concludes with challenges and research needs, emphasizing opportunities for computational and data-driven dynamic simulation approaches for multi-faceted applications.

## 1. Introduction

Food 3D printing, involving layer-by-layer food deposition (that is, additive manufacturing) is an emerging food customization and personalization technology in which materials are structured into preferred shapes. Customization is not limited to shape but can go well in terms of texture [1], nutrients [2], and nutraceutical ingredients [3]. Apart from developing novel product ranges, food 3D printing is regarded as an innovative approach towards waste valorization in the food industry and is also being recognized for its capabilities in developing sustainable food packaging solutions [4]. Among the various food printing approaches, extrusion-based techniques are more popular. They consider the rheological behavior and flowability of the material supply when sheared through a tiny nozzle. Further, there are different other material properties (such as textural and thermal behavior) and processing factors (such as printing nozzle height, diameter, printing rate, and nozzle movement rate) that govern the printing process, in addition to the complexity associated with post-processing and its impact on 3D printed constructs [5]. 

While copious research works are being undertaken in the field of food 3D printing, technologists often find several stages of the technology quite challenging. For instance, understanding the printability of a material is by itself a task—the material is subjected to different objective and subjective approaches to assess its behavior and applicability for a specific application. Similarly, the post-printing behavior is another aspect that is often poorly predicted. For example, fat content in the material supply can facilitate printability but might result in poor mechanical stability of the 3D-printed constructs. Given the need, just as in other food manufacturing processes, in the case of food 3D printing, attempts are being made to establish reliable and rapid approaches for process optimization, reduced time, reduced energy, improved process control, and reduced costs. 

Modeling and simulation are powerful tools, recognized for their strong capabilities to understand the underlying physics of different unit operations. They have been used in material selection, process condition optimization, improving process efficiencies, lowering costs, minimizing wastage, improving the life of processing equipment, and understanding the physio-chemical changes in foods during processing, in addition to other interesting applications. Models can be utilized in 3D printing processes to describe the phenomenon involved, through the understanding of the fundamental physics. Further, they can minimize multiple experiments by answering several ‘what if’ questions. For example, what if the size of the nozzle was 0.5 mm instead of 0.2 mm, or what if the starch content in the material is raised by 18%? In this context, modeling the 3D printability of different materials considers the thermomechanical characteristics of the materials [6], structural behavior [7], and the flow behavior of material supplies [8]. 

The printability of materials is presently evaluated by experimental determination of line width to nozzle diameter ratio, number of layers until the structure collapses, or the curvature of printed lines [9]. Considering food printing, printability estimation is time-consuming, and print fidelity is dependent on material properties and printing parameters. Modeling/simulation tools are evolving as a complement to experimental testing of these attributes.

Food printing, like other food processing operations, is inherently dynamic in nature, particularly in terms of changes in material supply properties before, during, and after printing, necessitating the optimization of these conditions to derive optimal and precise solutions [10]. Modeling and simulation tools can play a significant role in food 3D printing; for instance, to compute the optimized operating conditions that derive a process dynamic model with specifications for individual food material supplies, non-linear simulation models for parameter estimation based on the derived experimental datasets, and integrated process designs to determine the static system design variables as well as the processing conditions that can certainly consider cost-effectiveness. 

The development of predictive models is primarily focused on the requirement of evaluating food extrudability. To date, optimization approaches such as response surface methodology, which investigates the effect of various printing parameters on the printability of various foods, are being used to assess extrudability for 3D printing of foods. However, these approaches use statistical models based on the materials, limiting their applicability to a wide range of printing systems and material supplies.

Modeling or simulation tools or the requirement for associated approaches vary depending on the type of printing process and material supply used. For example, thermomechanical simulation tools are required for hot extrusion-based 3D printing to understand the 3D printability of foods under the application of heat and mechanical compression/shear. Printing of complex geometries requires a combination of printing methods (extrusion and injection) to establish flawless printing. This sometimes necessitates hybrid or integrated simulation approaches in which numerical and computational modeling tools are combined to improve the predictability of the model. This review details several modeling and simulation approaches that have been considered to predict the printing behavior and post-printing properties of material supplies. The article also emphasizes research requirements for developing effective predictive tools and integrating them into food 3D printing systems.

## 2. Summary of Studies on Modeling and Simulation of Food 3D Printing Processes

**Figure 1:**
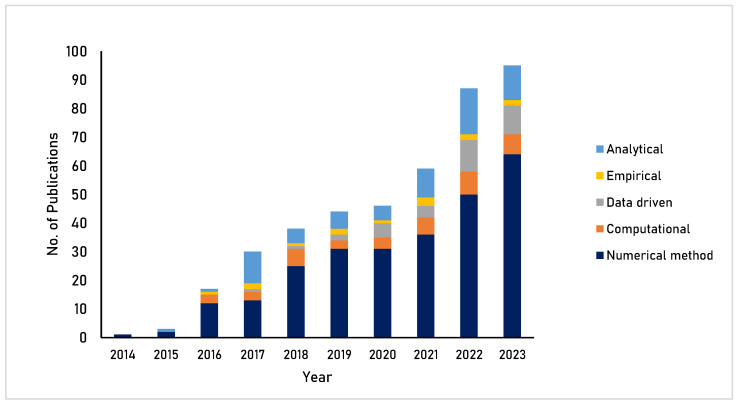
Year-wise trends in publications involving modeling and simulation applications in 3D food printing during the 10 years. Source: Online SCOPUS database (www.scopus.com) (accessed on 24 July 2023).

While food printing has gained prominence over the past two decades, applications involving modeling and simulation to improve 3D printing processes are even more recent. To have a better understanding of the rising thrust in terms of research in this area, a SCOPUS search with specific keywords was undertaken. Considering popular modeling approaches, different keywords were used: “numerical OR mathematical OR constitutive” for numerical modeling, “computational OR finite element OR finite volume” for computational modeling, “analytical” for analytical modeling, “neural network OR machine learning OR deep learning OR fuzzy OR artificial intelligence” for data-driven modeling, and “empirical” for empirical modeling, all in combination with “3D printing AND modeling AND simulation”. The year wise publication trends for the past 10 years (2014–2023) were assessed, revealing a count of 420 publications in total. Figure 1 shows the growth in terms of the number of publications, explaining the rising focus during the past 3–4 years. It is also evident from the literature survey that numerical approaches are more popular and data-driven modeling approaches are being increasingly used.

## 3. Methods of Simulation

In general, modeling/simulation tools are classified as mechanistic and data-driven approaches, with the former focusing on describing some or all mechanisms of a system with a few assumptions based on existing knowledge. Mechanistic models use phenomenological understanding to derive the physical attributes that contribute to defect formation in the additive manufacturing process. Data-driven models, on the other hand, extract information from large data sets in the form of linear or non-linear models for prediction. This section describes the mechanistic and data-driven modeling tools developed in the field of additive manufacturing.

### 3.1. Mechanistic Modeling

#### 3.1.1. Analytical Modeling

Analytical models are simulation tools that can be developed and have closed-form solutions. Analytical modeling is a simplified approach that allows for easier implementation with minimal computational effort. Analytical models are made up of simple relationships that connect regular structure geometries to their effective properties. The analytical approach can be subdivided into three categories: exact or closed-form solutions, approximate but closed-form solutions, and, more recently, numerical solutions [11]. Closed-form solutions represent simplified complex practical problems or attempts at solving real-world problems considering numerous assumptions, whereas, the numerical modeling approach provides the flexibility to simulate real-world processes considering several numerical techniques, which are described in the next section. These analytical models, however, are obtained with negligible effects of the process on product quality and are then fitted with experimental data to find the best fit analytical equation for the process [12]. Extrusion-based 3D printing has been represented in analytical models for the prediction of extrusion force [13], deformation and failure mechanism [14], elastic properties [15], and print fidelity. Numerous studies have used an analytical modeling approach to predict the mechanical properties of cellular structures in food 3D printing processes [16]. A novel analytical model was developed by Percoco et al. [13] for the prediction of extrusion force as a function of printing parameters. The geometrical parameters that defined the honeycomb were found to have a non-linear relationship with the effective Young’s modulus of the pectin-based samples. However, if these analytical models are not based on repetitive geometry, they produce laborious and complex expressions. This restricts the reliable representation of finite real-time material [16]. 

#### 3.1.2. Numerical/Computational Modeling

**Figure 2:**
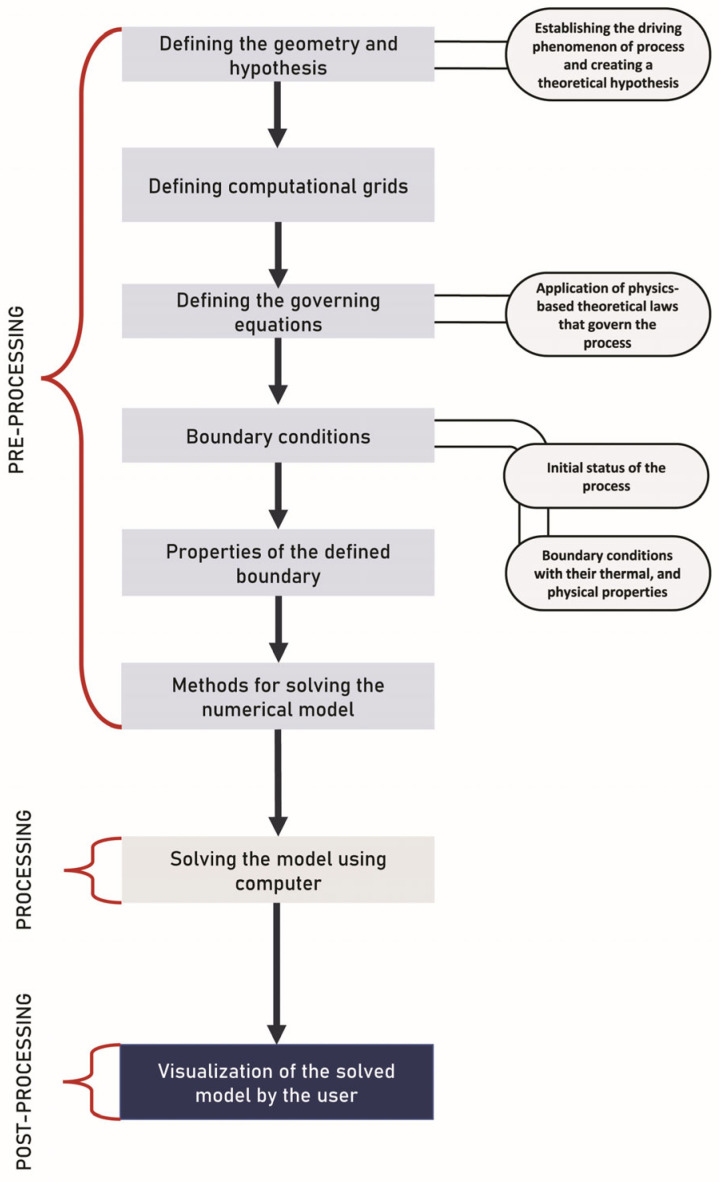
Steps involved in the mathematical simulation of a food processing operation within a defined geometry.

The numerical modeling approach ignores the reactions and mechanisms that occur during the process in order to understand a relationship between inputs (operating process conditions, product characteristics) and outputs (final quality attributes) using an experimental dataset, mathematical and statistical tools, and linear and/or nonlinear techniques. Mathematical simulation methods demonstrate the relationship between the properties of the material to be printed and the printability characteristics based on the experimental data sets obtained [17]. These models consider various governing equations, depending on the mode and type of simulation. These models can be used in process control systems as well as in evaluating complex processes using a practical approach. The numerical simulation approach also aids in the comprehension of real-time process monitoring in extrusion processing systems. The numerical simulation of the printing process in the virtual environment can increase the chances of successful and cost-effective printing. The steps involved in the mathematical simulation are described in Figure 2.

Mathematical simulation is a simplistic approach to representing the relationship between two attributes using the governing equations, and the model should consider several assumptions. For instance, in a study conducted on 3D printing of ceramic pastes, the material supply is incompressible and homogeneous and the properties such as elastic deform of viscoelastic paste, viscosity-dependent volumetric flow rate, and the friction between the paste and the extruder wall are considered negligible and ignored. The flow rate equilibrium equation of the extrusion process was developed. Considering food inks, mathematical simulation models can be used to display the fluid characteristics in an extruder by solving the model equations.

Mathematical models are established by researchers to find the relationship between the material supply properties and material supply composition. For example, Thangalakshmi et al. [18] have established a mathematical model between the rheological attributes of the material supplies in response to their ingredient composition, which can be used to define the most suitable ingredient mix composition with optimum storage, loss moduli, and flow stress. Various mathematical simulation tools have also been developed to evaluate the residual stress and deformation of a designed construct, which can then be used to modify the design and printing conditions based on the material supply properties [19]. These studies primarily employ the finite element method to analyze flow behavior, residual stress, total strain, swelling at the nozzle tip, and so on. According to Oyinloye et al. [20], the nozzle diameter is the most important influencing factor for the fluid properties in the flow field, as well as residual stress and total deformation of the printed surimi paste. 

The constitutive modeling approach is another type of simulation method for quantitative property prediction in complex geometries and loading conditions. Constitutive models, in general, capture the material’s intrinsic mechanical behavior [21]. These are primarily used for the quantitative evaluation of printed construct mechanical properties in relation to printing parameters such as printing angle, layer thickness, and so on. These constitutive models can be effectively used in finite element simulations, as reported by Wang et al. [22]. Anisotropic elastic-visco plastic-damaging behavior can also be predicted using constitutive modeling. For example, in a study using constitutive modeling, the damage is modeled using the effective stress concept in conjunction with the viscoelastic flow rule, which regulates the damage localization in selective laser-sintered (SLS) food [23]. Another study established a constitutive model to define the effect of moisture content on the viscoelastic behavior of SLS printed foods, as well as to comprehend their behavioral transition from brittle to ductile with increasing moisture content [24]. Constitutive models use a material’s viscoelastic response and fit a model to predict the effective properties of the material. Because different parameters are required to describe different loading conditions, the constitutive model’s predictive capability was limited. Jonkers et al. [21] used a finite element modeling tool to simulate the deformation behavior of brittle textured 3D-printed foods. The results showed that the constitutive model is suitable for predicting overall surface damage as well as localized damage when strain is applied, which is useful in predicting mechanical deformation behavior. 

Many researchers have relied substantially on mathematical simulation to better understand the extrusion process in real time. This method has been used to simulate the 3D printing process, explaining the non-uniformity of velocity distribution in a flow channel and the resulting jet expansion during the printing process. Mathematical simulation models were used in food 3D printing to simulate the effect of different material properties and process parameters on the velocity, local shear rate, and pressure fields in the flow channel [25]. A numerical model was used in another study to reveal the distribution of the flow field during the extrusion process of power-law fluid in an extrusion-based 3D printing. During the printing process, the distribution of velocity, local shear rate, viscosity, and pressure in the flow field was measured using a numerical simulation method [26]. The numerical simulation approach has also been investigated for other 3D printing applications such as fracture behavior in 3D printed constructs. 

Computational fluid dynamics (CFD) is a computer simulation method for characterizing fluid flow behavior in specific geometries with boundary conditions. CFD is increasingly being used by researchers, system manufacturers, and process engineers in the food and bioprocess industries to study the flow behavior and efficiency of process equipment such as baking ovens, refrigerators, spray dryers, and 3D printers. In food 3D printing, computational simulation is used not only to predict fluid behavior, but also to determine complex flow characteristics, chemical reaction kinetics, mechanical movements, and structural deformation behavior. CFD technology was used in the 3DP process to identify the critical parameters that dictate the shape of the printed product as well as to find the best material deposition strategy. Thus, CFD has contributed to lower production costs, higher product quality, and increased productivity (i.e., printing speed). The incorporation of computation modeling into 3D printing has accelerated the understanding of fluid mechanics within device testing in research and development. Computational modeling approaches can be classified as mesh-based and mesh-free methods. 

Finite element modeling (FEM) is a mathematical modeling approach that allows for the analysis of an infinite number of variations in structure designs while incorporating local effects such as imperfections. Furthermore, by combining FEM with imaging techniques such as micro-computed tomography, the actual geometry of specific samples can be modeled. It is a mesh-based method in which the elements are subdivided into elements to analyze the mechanical properties, deformation behavior, and other characteristics of food 3D printing.

Mesh-free methods, such as smoothed particle hydrodynamics (SPH), are Eulerian picture simulation tools that can be used to evaluate food deposition simulations, which have also been studied for food 3D printing. A moving set of particles discretizes the governing equations in SPH. It has a highly adaptive nature, is well-suited to treating large deformations, moving interfaces, or free surfaces, and can provide the time history of field variables [27]. SPH has been used to model free surface behavior, interactions with solid surfaces, and fluid–solid interactions, as well as multiphase or particulate flow, fluid mixing, and turbulent flow. The fluid is discretized as spheres in these methods, and the interaction between fluid molecules is defined using the molecular dynamics approach. This method is primarily used to determine the post-print stability of spherical molecules when they are printed. In a study conducted by Makino et al. [28], a proportional relationship was obtained between the fluid viscosity and the shape retention ability. Discrete element modeling is another mesh-free approach to quantify the changes in a discontinuous phase of a sample. 

### 3.2. Data-Driven Modeling

Data-driven modeling approaches use measured data to determine the regression of model parameters to fit the input–output behavior. Various mathematical representations (such as artificial neural networks, fuzzy logic, and so on) and mathematical techniques (such as principal component regression, support vector analysis, and so on) are used in this approach to understand process behavior from datasets [29]. These data-driven models are used to instantly determine an unmeasured variable based on real-time data. Researchers investigated a variety of data-driven computational modeling tools to simulate the processing conditions of 3D printing in foods. Machine learning approaches have been successfully used to predict the printability of various non-food materials. Data-driven modeling is a method in which model components are dynamically injected into the model based on data from external experimental systems. Grey box modeling is a hybrid modeling approach that combines mechanistic and data-driven tools to maximize the predictability of the complex relationship between material properties and printing parameters. This section investigates various data-driven modeling approaches for predicting printing behavior in food printing applications.

#### 3.2.1. Artificial Neural Networks

Artificial neural networks (ANN) in food 3D printing applications are used in various applications such as process monitoring, designing, and the correlation between process parameters and final characteristics of the obtained component. In 3D food printing, artificial neural network models use the physicochemical properties or other material supply properties as the input signals which can further be analyzed to obtain the printing conditions. In a study by Guo et al. [30], LF-NMR signals which were measured to analyze the water distribution and condition of polysaccharide gels were used as the input signal for the Backpropagation ANN for the prediction of piston pressure and printing scores. Ten hidden layers were used for the best fitting performance and the results suggested that the piston pressure values were fitted well with a correlation coefficient of 0.951 during the testing phase. 

#### 3.2.2. Machine Learning

In the unsupervised learning approach, various clustering algorithms have been used to analyze the relationship between printability and material supply properties. Hierarchical cluster analysis was used in a study to characterize the printability of surimi gels at different extrusion conditions. These clusters are reported as dendrograms based on their rheological properties [31]. In another study, it was reported that regarding the 3D printability of surimi, the starch system was closely related to the type and addition content of starch and the water and rheological properties [32]. Another study has presented a mind-controlled 3D food printer that translates the brain signals obtained by EEG in real-time into the emotional valance and arousal levels using a machine learning algorithm and determines the shape and size of the food to be fabricated by the food printer. The approach used the Gradient Boosting Decision Tree classifier approach to extract the feature scores of the variables. Other machine learning approaches, such as deep learning and artificial intelligence, have the potential to be used in real-time decision-making systems for food 3D printers.

#### 3.2.3. Fuzzy Systems

Fuzzy inference systems (FIS) were developed based on fuzzy logic (FL) to provide a method for expressing blurry attributes and to allow the integration of data and information from subject matter experts. As a synergic hybrid intelligent system, the adaptive neuro-fuzzy inference system (ANFIS) has emerged. It combines the fuzzy logic systems’ (FLS) human-like reasoning style with the learning and computational capabilities of ANNs. ANFIS systems are used in fused deposition modeling 3D printing systems to evaluate surface roughness, build time, and compressive strength as printing parameters change [33]. To date, no studies have investigated the use of ANFIS architecture for estimating textural aspects in food printing. 

### 3.3. Integrated Modeling Approaches

The synergistic approach of using mechanistic modeling with a data-driven machine learning approach provides important quantitative correlations, which can reduce the number of experiments required to improve the quality of the additive manufacturing process. An integrated machine learning and mechanistic model was developed by Du et al. [34] in which the mechanistic modeling approach was used to determine the defects and the data drawn from the mechanistic model were provided as the input data for the machine learning algorithm, which has shown to be a potential tool to reduce common defects and other complex engineering problems during additive manufacturing of alloys. The data generated by the mechanistic model can be effectively used by machine learning algorithms to establish the relationship and optimize the processing conditions. The integrated modeling approach has also been demonstrated as a feasible modeling approach for the field of additive manufacturing. A conceptual framework integrating a mathematical modeling approach with machine learning and statistical analysis methods was demonstrated to optimize the process parameters of powder bed fusion additive manufacturing [35].

## 4. Applications of Simulation Tools in 3D Food Printing

### 4.1. Optimization of Material Supply

The ingredients used for the preparation of 3D food printing material supplies must be chosen carefully as they have a significant influence over printability and overall product quality [5]. The behavior of the material supplies under different printing parameters suggests its printability. The common approaches for evaluating the suitability of 3D printing material supply include performing rheological characterization such as static and dynamic rheological tests and printability assessment tests such as line tests, cylinder tests, and extrusion tests. The material supplies possessing tunable mechanical strength and appropriate shear thinning properties were identified as suitable for 3D printing [36]. In food applications, the diversity of the material supply ingredients and their behavior makes the characterization process more strenuous. Under such conditions, different modeling and simulation tools can help in predicting the behavior of material supplies for assessing their suitability for 3D printing.

Among the different 3D printing technologies available, extrusion-based 3D printing is most used in food processing. During extrusion, the material is sheared and pushed out of the nozzle. Studies have reported the use of modeling methods for simulating the complex process of extrusion. This includes different approaches such as data-driven modeling using ANN [37], the deterministic mathematical model for flow behavior and bubble growth dynamics [38], numerical simulation for the extrusion of pasta dough [39], and many more. Based on different studies on extrusion-based 3D food printing it can be said that the printability strongly depends on the rheological, thermal, and gelling properties. Different approaches to modeling and simulations have been reported to study these properties and predict the 3D printing behavior of various material supplies. Recently, a data-driven modeling-based approach was used to predict the extrudability of material supplies. A unique combination of material supplies with varying rheological properties with different printing conditions was printed. In order to measure the mode width, line height, and width consistency of the printed filaments, image analysis was undertaken. Further, regression models were used to predict the parameters using rheological properties and printing conditions as input, and further classification models were used to categorize the print as acceptable and unacceptable. With the application of a random forest algorithm, the prediction of extrudability based on rheological properties and printing parameters can be undertaken with moderate to high extrudability [40]. 

To throw more light on the flow of material through the nozzle, Yang et al. [25] reported a numerical simulation technique for simulating the flow of material in a flow channel as a function of material properties and 3D printing process parameters. It was observed that the inlet volume flow rate significantly affected the velocity and shear velocity flow fields, whereas material viscosity and nozzle diameter strongly affected the pressure fields. Nozzle diameter had the greatest impact on pressure fields inside the flow channel. Based on the simulations and actual experiments, it was also observed that the material tends to undergo swelling post-extrusion which can be overcome by using a 90% filling rate. 

**Figure 3:**
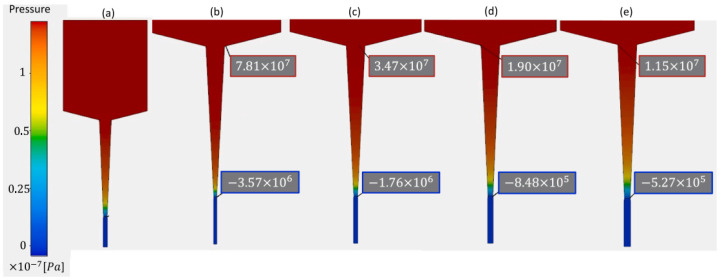
Visualization of pressure distribution: (**a**) flow field description (**b**) 0.6 mm, (**c**) 0.8 mm (**d**) 1.0 mm, and (**e**) 1.2 mm nozzle diameter “Reprinted/adapted with permission from Ref. [20]. Copyright © 2022. Elsevier”.

**Figure 4:**
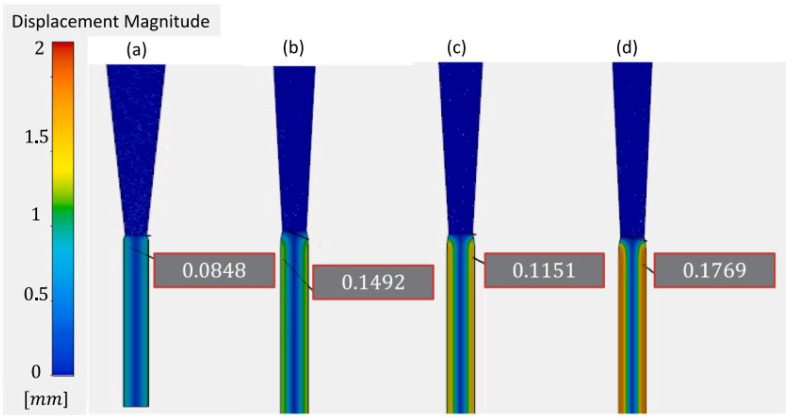
Visualization of die swell as a result of different nozzle diameters, (**a**) 0.6 mm, (**b**) 0.8 mm, (**c**) 1.0 mm, and (**d**) 1.2 mm. “Reprinted/adapted with permission from Ref. [20]. Copyright © 2022. Elsevier”.

Another important aspect related to successful printing is the stability of 3D-printed layers during and after printing. The stability strongly depends on several phenomena such as residual stress, die swell, and deformation during extrusion, which are, in turn, controlled by printing parameters such as pressure and nozzle diameter. In a study on surimi gels, Oyinloye et al. [20] used the finite element method to visualize the aforementioned phenomenon during extrusion-type 3D printing. Based on the rheological properties, a surimi paste with 82% moisture content was found to be optimum for 3D printing. Figure 3 and Figure 4 indicate the pressure distribution and die swell as a result of different nozzle diameters, respectively. A lower nozzle diameter resulted in higher pressure in the chamber because a smaller nozzle diameter creates restrictions for the flow of the material. Furthermore, pressure is strongly linked to the die swell after the extrusion of the paste. As the pressure increased with a reducing nozzle diameter, the die swell ratio also increased, which can be attributed to the viscoelastic nature of the material supply. An increase in the pressure increases the elastic potential energy in the material supply undergoing extrusion, which plays a major role in die swell, which further significantly influences the deformation behavior of 3D printed materials. 

**Figure 5:**
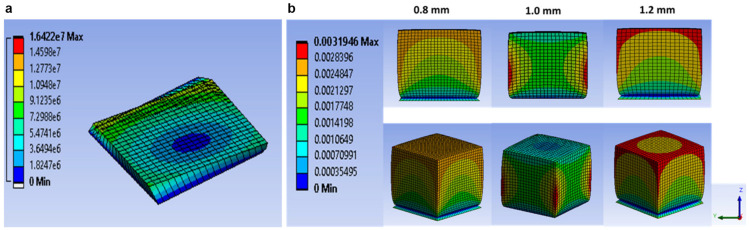
Simulation of deformation in an uncontrolled environment (**a**) and controlled environment at 47 ± 5 °C (**b**) with different nozzle diameters. “Reprinted/adapted with permission from Ref. [41]. Creative Commons CC-BY license, Copyright © 2021”.

Apart from other printing variables, the effect of ambient temperature during the deposition of the 3D printing material supply is also a crucial parameter, particularly in connection with the thermal behavior such as gelatinization. Oyinloye and Yoon [41] studied the effect of ambient temperature among other variables on the deposition and slumping behavior of rice paste. Rice paste printed at ambient temperature showed structural breakdown and slumping behavior owing to shear stress and weak elastic characteristics. Figure 5a,b shows the simulated deformation in 3D printed constructs printed at an uncontrolled environmental temperature (27 ± 2 °C) and a controlled temperature (47 ± 5 °C) with different nozzle diameters, respectively. This suggests that improvement in the elastic properties of material supply is owing to the gelatinization of starch. Additionally, temperature distribution and subsequent stress and deformation were found to be dependent on nozzle diameter. Before modeling and simulation of such a complicated phenomenon, the 3D printing conditions can be optimized for better printing precision and shape retention. Similar applications of modeling and simulation in 3D food printing are reported in Table 1.

### 4.2. Design of 3D Printer

**Figure 6:**
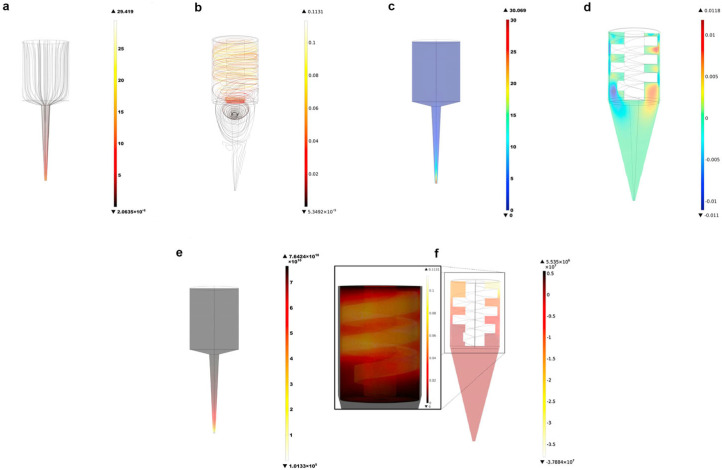
Velocity profile of syringe−type (**a**) and screw−type (**b**), longitudinal velocity distribution in syringe−type (**c**) and screw−type (**d**), and pressure distribution in syringe−type (**e**) and screw−type (**f**) 3D printers. “Reprinted/adapted with permission from Ref. [49]. Copyright © 2019, Elsevier”.

Three-dimensional food printers are available in a variety of designs. Though extrusion-based 3D printing is the most common, other types such as selective sintering and binder jetting are also used. Moreover, in the extrusion type, different configurations involve the use of a screw, piston, or pneumatic system for pushing the material supply. Apart from these, air pressure-assisted screw configuration is also commonly reported. The choice of 3D printer configuration depends on the material supply properties, complexity, and ease of operation. Mathematical and computational modeling can easily predict the behavior of the material in different configurations of 3D printers. For instance, Guo et al. [49] compared the fluid flow patterns in two different configurations of extrusion 3D printing; namely, injection-based and screw-based. Printing experiments revealed the printing profile, whereas, computational modeling and simulation provided insights into the fluid flow patterns. The simulation revealed that syringe-type 3D printing showed relatively simpler flow profiles compared to screw-based 3D printing. The velocity profile of the screw-type configuration showed higher velocity around the screw contradictory to the syringe-type where maximum velocity was observed at the outlet of the nozzle (Figure 6a,b). Furthermore, the longitudinal velocity distribution (Figure 6c,d) indicated similar results. Higher longitudinal velocities were found under the screw thread and a backflow of material can be visualized between the gap of flight and the barrel wall. Further, the simulated pressure distribution in both printing combinations is visualized in Figure 6e,f. The syringe configuration showed highly homogenous pressure distribution with a sharp decline near the nozzle outlet which is desirable for smooth extrusion. On the other hand, in the screw-type configuration, pressure distribution was relatively uniform in the nozzle area compared to the barrel. The highest pressure was observed at the bottom of the barrel, which can result in a backflow when pressure exceeds the threshold value. A high shear rate and back-flow of material was reported in the screw-type 3D printer, which suggests its non-suitability for high viscosity materials. 

With such modeling and simulation studies, further research can be taken up to understand the design limitations and improvement in the same. Nevertheless, the back-flow issue can be overcome by minimizing the gap between the screw flight and the barrel wall. With such modeling and simulation studies, further research can be taken up to understand the design limitations and recommendations can be provided for improved design of 3D printers. Another interesting application involved the use of computer vision and a feed-forward control approach for improved printing accuracy. A side-view camera films the extrusion process and determines the extruded filament thickness through image processing. Based on these data as input, the feed-forward control mechanism adjusts the nozzle movement speed to maintain printing accuracy. The major advantage is the flexibility of this system which allows it to be integrated with most extrusion-based 3D food printers for initial calibration purposes. This can effectively eliminate the strenuous trial-and-error approach of 3D printing optimization [50].

**Figure 7:**
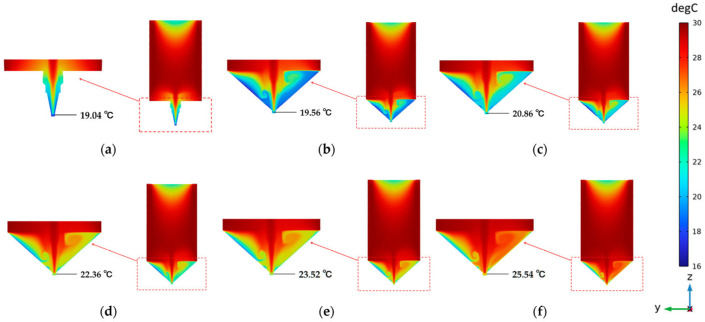
Visualization of steady-state temperature distribution in initial (**a**) and improved nozzle design with different water temperatures (**b**–**f**). “Reprinted/adapted with permission from Ref. [52], Copyright © 2023”.

Apart from different design aspects such as the configuration of 3D printers, the design of nozzles, and many more, the temperature is a significant parameter affecting the printing performance of material supplies. This is more commonly observed in the case of gel-like materials (gelatin, agar) and fat-rich materials such as chocolates [51]. The mechanical and rheological properties of these materials significantly depend on temperature due to their melting behavior. Therefore, precise control over temperature during 3D printing is essential for excellent printing precision and stability. In the case of the 3D printing of gelatin, it was observed that the convective heat transfer between the surrounding environment and nozzle resulted in a thermal gradient which negatively influenced the accuracy of printing. To address this, an improved water circulation design was developed to prevent heat loss and more precise control over the nozzle temperature. The effect of water temperature on the heat temperature profiles was evaluated through computational modeling using the finite element method to arrive at the optimum conditions. Figure 7 represents the temperature distribution around the initial nozzle and the improved water circulation nozzle with different water temperatures. Based on the simulation results, it was observed that integration of the improved design of the nozzle resulted in a limited temperature drop and a much gentle temperature gradient compared to the initial nozzle configuration. Compared with experimental data, the nozzle domain temperature should be 25 °C to obtain optimum extrusion. Due to the simplistic approach to modeling the heat transfer process, the simulated temperature deviated from the actual temperature. Nevertheless, the work offers valuable insights into temperature-dependent 3D printing of low-viscosity gels [52]. Temperature distribution in the 3D printed constructs also has been visualized to study the model firmness. 

### 4.3. Texture Modification (Hardness-Targeted Designs)

The selection of a material supply with tunable mechanical strength and suitable shear-thinning properties can assist in texture modification. Simulation tools are also used in the 3D printing of foods to produce structural food materials with specifically targeted textural attributes. A major advantage of 3D food printing technology is the ability to develop personalized and customized foods by altering the micro/macrostructure of foods [53]. Apart from the shape, size color, and nutritional composition, 3D printing holds the potential for tailoring the texture of foods. The impact of material supply properties, 3D printing parameters, and post-processing conditions is also elaborated. In this context, the role of computational modeling and simulation is also highlighted. In the case of non-food applications, numerical approaches have been employed for predicting the elastic properties of fiber-reinforced polymer structures [54] and the mechanical behavior of 3D-printed alginate scaffolds [55]. 

In a study conducted by Fahmy et al. [56], a parametric design approach was used for the prediction and digital design of hardness. Finite element analysis was used to fit the parameters and to derive a generalized hardness design formula. It is also important to characterize the deformation behavior through the 3D printing process to control the food texture perception and subsequent designing of texture-modified foods. phenomenological foam model (PFM) is used to evaluate the stress–strain behavior of the 3D-printed cellular structures. In such conditions, the finite element modeling approach was used to simulate the developed stress, associated deformation behavior, and hardness of the designed food configurations. Hardness targeted—PFM modeling enables the design of printing attributes with respect to the required specific hardness values. A mathematical equation was derived to define the relationship between the specific hardness values (*H_unit cell_*) of printed constructs and their properties such as effective compression area (*A*), design porosity, (*P*), and Young’s modulus (*E*) (kPa) (Equation (1)) [57].

(1) $$H_{unit\;cell\;}=-1.23+\left(8.24\times 10^{-5}\right)\;A+\left(7.1\times 10^{-4}\right)+\left(1.15\times 10^{-8}\right){\;A}^{2}+\left(1.2\times 10^{-6}\right)\;EPA$$

**Figure 8:**
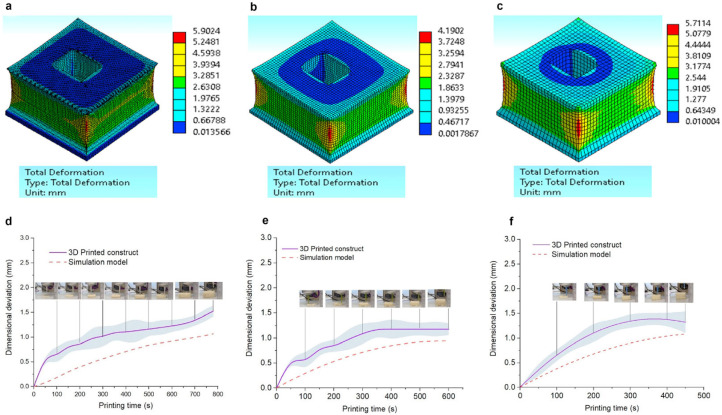
Total distortion profiles during 3D printing with 1.24 mm (**a**), 1.38 mm (**b**), and 1.56 mm (**c**) nozzle diameters and comparison of simulated and experimental width deviation with different nozzle diameters of 1.24 mm (**d**), 1.38 mm (**e**), and 1.56 mm (**f**). The grey shadow part represents error band. “Reprinted/adapted with permission from Ref. [58]. Copyright © 2023, Elsevier”.

The final texture and mechanical properties of 3D-printed foods are strongly dependent on their microstructure and ultimately on the printing conditions and ingredients used. In a work on brittle foods prepared by selective laser sintering, Jonkers et al. [21] combined constitutive modeling and the finite element method to predict the mechanical properties of 3D-printed porous, brittle foods. In another work, different infill patterns and densities were used to develop 3D-printed sweetmeats with personalized textures. To achieve this, first, the material supply comprising heat acid coagulated milk semi-solids, whey protein isolates, and maltitol was characterized using rheological tests to assess its suitability for 3D printing. Further, a finite element-based approach was used to assess the effect of printing parameters by visualizing the stress distribution and distortion profile. Figure 8a–c represents the distortion profile of the 3D printed constructs with 1.24 mm, 1.38 mm, and 1.56 mm nozzle diameters, respectively. It has to be noted that the distortion in 3D-printed constructs primarily arises from the post-printing tension. With the increase in the nozzle diameter, projected distortion decreases, which underlines the necessity of the optimum flow for successful 3D printing. The predicted distortion results were validated by comparing them with experimental values obtained after each layer of printing (Figure 8d–f), which suggests the suitability of the model for the prediction of the shape distortions. In order to predict the effect of different printing conditions on the texture of sweetmeats, a predetermined experimental model coupled with multivariate data analysis models was followed [58].

### 4.4. Post-Printing Stability

Typically, the 3D-printed food constructs are subjected to some form of post-processing such as drying, frying, steaming, microwaving, and so on. The success of the 3D printing of foods depends not only on the printing precision but also on the way 3D printed constructs react to post-processing. Theagarajan et al. [59] evaluated the effect of different post-processing methods on 3D-printed rice constructs. Computational modeling and simulation tools can offer an alternative to such trial-and-error approaches for predicting dimensional changes and stability. In the case of 3D printing of ceramic pastes, factors such as non-uniform drying and subsequent effects such as cracks and deformation limit their applicability. To address this, a steady-state equilibrium equation for the extrusion process was derived for a complete theoretical understanding of the process. The model considered all realms including rheological behavior, extrudability, shape-holding capacity, and drying kinetics of post-processing. Further, the study of the drying kinetics of 3D printed constructs revealed the different forces and stresses acting that are responsible for the deformation. Based on these kinetics and finite element simulations, an in situ hot air flow drying device was designed which can be mounted along with a nozzle and ensure uniform drying of the printed constructs. A novel modeling-based approach of post-printing shape retentions was observed to be more successful than the conventional approaches such as deposition in a freezing environment and use of removable support structures [60].

Yet another advanced version of additive manufacturing is 4D printing, which refers to the change in 3D printed materials in terms of shape, properties, and functions over time in response to external stimuli [61]. Programming the structure of a 3D-printed construct to achieve the desired transformation as a result of a specific stimulus is of great importance here. Modern modeling and simulation tools can effectively predict the behavior of 3D printed materials as a result of external stimuli. This information would be valuable while designing a 4D printed food. In a work on shape memory polymers, Serjouei et al. [62] developed sandwich structures for reversible energy absorption applications. A finite element approach was used to accurately predict the compressive behavior, energy absorption capacity, and shape recovery of these materials. Similarly, these approaches could be effectively applied to predict the behavior of 3D-printed food materials under applied stimuli.

## 5. Challenges and Directions for Future Research

Though the modeling/simulation tools can bring commercial feasibility to the 3D food printing systems by predicting complex changes in geometry and material supply, the following research-related challenges need to be addressed shortly.

- Dynamic changes in material properties and process conditions: biomaterials often undergo dynamic changes in their properties upon the application of shearing or compression during the process. This limits the applicability of using mathematical predictive models, as the decision-making becomes complex due to the process dynamics. In such cases, the dynamic changes in the material supply properties that affect the processing conditions need to be considered in the model to minimize errors in decision-making.
- Uncertainties in decision-making: the behavior or processes defined within the model and the assumptions made in the model determine the uncertainties in any decision-making process involving modeling/simulation tools. Thus, it is important to fully understand the system behavior and underlying physics to represent a 3D printing system as a real-world system, minimizing the assumptions that contribute to such uncertainties.
- Scaling up the technology: material supply properties, food supply chain factors, and a lack of knowledge about high-capacity 3D printers all influence the widespread adoption of 3D printing technology. Simulation tools will be used to determine the required rate of shear or compression on a specific type of material supply. This would aid in the development of a printer capable of printing materials based on their basic material supply properties. Integrating 3D printing systems with real-time human–computer interaction tools has the scope of being a potential solution for scaling up the technology. For example, Ninyawee et al. [63] presented the concept of integrating a brain–computer interface with 3D food fabrication systems by training the system using a machine learning classifier-based emotion recognition system. These machine-learning models have presented about 59.87% valence accuracy and 61.10% arousal accuracy during real-time classification. In addition, business models are considered to assess the financial viability of the technology. These approaches need to be investigated further in order to optimize a viable solution for commercializing the technology.
- Computational challenges: often, simulation studies handle huge volumes of data and demand high computational resources. This also includes the demand for highly skilled professionals to handle them. For instance, a strong understanding of the underlying phenomena is mandatory to ascertain the governing equations, solver settings, and a range of other test conditions. The potential of modeling and simulation tools can be potentially improved by established technologies such as big data, artificial intelligence, cloud computing, the Internet of Things, and supercomputer architectures. Integrating artificial intelligence–machine learning technologies with 3D printing will be useful in developing personalized foods according to individual consumer needs [64]. The applicability of these technologies to develop simulation tools for complex operations in 3D printing processes can be explored.
- Assumptions: models developed to mimic real-world systems frequently make numerous assumptions to create a simplified form of the model that can be analyzed without the use of high-end software/computers. This can reduce the precision of the model. Even if the assumptions cannot be eliminated, the assumptions made on major influencing factors must be identified and eliminated to improve the model’s prediction efficiency.
- Model reusability and adaptability: the models developed need to have the flexibility of reuse for similar geometries and printing conditions. This can be achieved by developing integrated models that can produce reliable results. The choice of working with 2D or 3D models will always have a significant impact—the processes cannot be oversimplified or underestimated. Also, while the developer can work on various CFD codes and modeling tools, the end-user would find it beneficial when these concepts are translated to conveniently usable software with suitable user interfacing.
- Model validation: all models developed to simulate the 3D printing process must be validated, either experimentally or by feeding an unknown input data set to a data-driven modeling algorithm to derive the output data. The validation shall provide the model’s prediction efficiency and define the model’s adaptability in real-world systems.

## 6. Conclusions

Three-dimensional food printing is a complex unit operation and involves a strenuous trial and error approach for the assessment of printability, optimization of material supply and 3D printing conditions, and prediction of post-processing behavior. To overcome this challenge, various modeling and simulation approaches have been explored recently. Among these, computational models are gaining popularity due to their capability to simulate process dynamics within a constrained boundary. They can predict the velocity, flow, pressure, and shear distribution during 3D printing in real-time. Real-time visualization of residual stress and die swell enables the prediction of the stability of 3D printed constructs. Despite several advantages, there is a scope for improving the performance of modeling approaches by reducing the number of assumptions. Furthermore, the use of hybrid approaches combining different modeling methods can help in the better prediction of the behavior of materials undergoing 3D printing. Four-dimensional food printing is an emerging area in which the 3D printed constructs undergo self-modification in response to an external stimulus. Modeling and simulation tools will be of key importance for programming the 3D-printed foods to attain desired modifications upon exposure to a stimulus.

## Figures and Tables

**Table 1:** Applications of modeling and simulation in 3D printing of foods.

Application	Material Supply	3D Printer Type/Configuration	Modeling Approach	Software Used	Key Findings	Reference
Optimization of 3D printing parameters	Ketchup, chocolate pudding, peanut butter, mayonnaise, jam	Piston-type extrusion	Mathematical Modeling	-	Extending the application of the modified Hagen–Poisuille equation to thixotropic materials; A 3% deviation between the experimental volumetric discharge and the value obtained by the theoretical equation	[42]
	Heat acid coagulated milk semisolids and polyol matrix	Syringe-type extrusion	Finite element method	ANSYS POLYFLOW® (ver. 18.1)	A correlation between printing parameters and flow field characteristics was established; A mathematical model to predict shear rate, pressure, and velocity inside the flow channel for the prediction of other printing parameters	[43]
Assessment of printability of material supply	Black rice, Job’s tear seeds, mung bean, brown rice, and buckwheat	Piston-type extrusion	Computational fluid dynamics (Finite element method)	ANSYS POLYFLOW ® (ver. 18.1)	Computational simulation coupled with the Bird–Carreau model effectively represented the flow behavior of grains under extrusion; Simulated results indicate piston pressure is the criteria for evaluation of printability	[44]
	A combination of wheat flour, sugar, butter, water, and potato granules	Piston-type extruder	Computational fluid dynamics (Finite element method)	POLYFLOW	Pressure in the flow field is directly proportional to the consistency index and indirectly, non-linearly proportional to the flow behavior index	[26]
	Potato starch, sodium alginate, xanthan gum, water		Computational fluid dynamics (Finite volume method)	OpenFOAM (ver. 18.06)	Lower velocity at the junction of cylinder and nozzle which indicates accumulation of material; Visualization of dynamic changes in rheological properties of material supply during printing	[45]
	Sodium alginate and pea protein	Extrusion	Finite element method	COMSOL ® (ver. 3.5)	Temperature field distribution, residual stress, and total deformation were dependent on deposit thickness; With additive layer manufacturing simulation, optimum printing conditions can be achieved efficiently compared to standard printing	[46]
Prediction of textural properties	Cookie dough		Finite element method	COMSOL® (ver. 4.3)	Establishment of the relationship between Young’s modulus and honeycomb structure parameters of 3D printed cookies; Wall thickness, cell size, and overall porosity emerged as tunable design parameters	[47]
Prediction of post-processing behavior	Yellow peach-buckwheat paste	Syringe-based extrusion	Finite element method	COMSOL® (ver. 4.3a)	The simulated surface temperature distribution agrees well with the one obtained by the thermal imager; Formation of the hot spot at the gap between the petal and stamen of the model results in a large strain responsible for 4th-dimensional change; Layer-by-layer structure of 3D printed construct must be taken into consideration while building a model for simulation	[48]

## Data Availability

Not applicable.
